# *Staphylococcus aureus* bacteraemia cases at Helen Joseph Hospital

**DOI:** 10.4102/sajid.v39i1.626

**Published:** 2024-05-27

**Authors:** Mithra John, Lauren Richards, Jeremy S. Nel

**Affiliations:** 1 Department of Internal Medicine, Faculty of Health Sciences, University of the Witwatersrand, Johannesburg, South Africa; 2 Division of Infectious Diseases, Department of Internal Medicine, Faculty of Health Sciences, University of the Witwatersrand, Johannesburg, South Africa

**Keywords:** *Staphylococcus aureus*, bacteraemia, infectious diseases, inpatients, Helen Joseph Hospital

## Abstract

**Background:**

*Staphylococcus aureus* bacteraemia (SAB) is associated with a high mortality. Data on SAB cases in South Africa (SA) are limited.

**Objectives:**

This study aimed to establish the demographic profile, risk factors and complications of patients with SAB in a tertiary inpatient setting.

**Method:**

We conducted a retrospective record review of inpatients above the age of 13 with SAB from October 2015 to November 2022 at Helen Jospeh Hospital (HJH) in Gauteng, SA.

**Results:**

A total of 126 patients with SAB were reviewed. The case fatality ratio among these patients was 20.6% (95% confidence interval [CI]: 13.9–28.8); this was similar for methicillin-sensitive *S. aureus* and methicillin-resistant *S. aureus* (*p* = 0.154). Almost half (49.2%) were community acquired, and these were chiefly associated with skin and soft tissue infections (45.2%), while most healthcare-associated community-acquired infections (18.3%) and nosocomial-related infections (32.5%) were associated with short-term venous catheterisation (40.6%). The most common risk factors for acquiring a SAB were prior hospitalisation in the last 90 days (27.8%), the presence of an invasive device (26.2%) and receipt of haemodialysis (15.1%). Having hypertension (adjusted odds ratio: 5.55 [95% CI: 1.31–23.55]) and being recently hospitalised (adjusted odds ratio: 11.88 [95% CI: 1.84–26.99]) were associated with statistically significant increased odds of death.

**Conclusion:**

SAB-associated all-cause mortality remains high in a middle-income tertiary hospital setting, albeit with a case fatality ratio comparable to that seen in high-income countries.

**Contribution:**

Our study suggests that acceptable outcomes are achievable in tertiary middle-income settings provided there is access to resources including infectious diseases consultation, echocardiograms and basic infection control practices.

## Introduction

*Staphylococcus aureus* bacteraemia (SAB) is a major global health concern with an annual incidence of 50/100 000 population and a case fatality ratio of 20% – 30% in high-income countries despite effective antibacterial therapies and source control strategies.^1^ Local South African (SA) data have suggested a range of SAB-associated mortality ranging from 16% to 41.8%.^2,3,4^ *Staphylococcus aureus* bacteraemia risk factors that are frequently identified are intensive care unit (ICU) admission, organ dysfunction, and older age, and these have also been linked to higher mortality.^2,3,5^

Meta-analyses have additionally shown that methicillin-resistant *Staphylococcus aureus* (MRSA) bacteraemia is associated with an increased mortality compared to methicillin-sensitive *Staphylococcus aureus* (MSSA).^6^ In a retrospective review of 442 SAB cases at three sentinel sites in SA, 36% of patients had an MRSA infection. A longer hospital stay, hospitalisation in the last year, human immunodeficiency virus (HIV) infection and antibiotic use in the previous 2 months were independent predictors of mortality.^5^

Most local SAB studies have focused on MRSA bacteraemia. There have been very few studies assessing the risk factors, mortality and epidemiology of SAB cases in SA in general, and the few studies that do date to as far back as 2002. The impact of HIV on SAB, modes of SAB acquisition and complications of SAB have not been well described in the sub-Saharan setting.

We conducted a retrospective review at a SA tertiary hospital to describe the demographics, underlying comorbidities and outcomes of SAB patients. We further explored factors associated with disease acquisition, mortality and SAB complications.

## Research methods and design

We conducted retrospective cohort study of patients with SAB treated at Helen Joseph Hospital (HJH), in Johannesburg, SA. The hospital, a 600-bed tertiary academic facility, mainly serves urban and peri-urban areas, including informal settlements.

We included all inpatients at HJH over 13 years of age who had a pure growth of *Staphylococcus aureus* in one or more blood cultures over an 8-year period from October 2015 until November 2022. Patients were identified through records from the Division of Infectious Diseases at HJH, which is routinely alerted by the hospital’s microbiology department of all SAB cases from the hospital. Thereafter, data were supplemented from inpatient hospital records. Patients were excluded if their hospital records could not be found or if their outcome data (i.e. whether the patient died prior to discharge or were alive at hospital discharge) were missing. An attempt was made to obtain each patient outcome by searching the laboratory database to determine survival in cases where the hospital records were unobtainable. For each patient, positive *S. aureus* blood cultures were considered as part of the same episode if they occurred within a single hospital admission.

*Staphylococcus aureus* bacteraemia was defined as the presence of one or more blood cultures positive for *S. aureus.* Outcomes were defined as ‘deceased’ or ‘discharged’ during their in-hospital admission period. *Staphylococcus aureus* bacteraemia was classified as community acquired if *S. aureus* was first cultured from blood obtained within 48 h of admission. Healthcare-associated community-onset *S. aureus* bacteraemia was defined as *S. aureus* cultured from blood obtained within 48 h of admission that occurred in the presence of one or more of the following healthcare risk factors:

- The presence of a long-term invasive device at time of admission.
- A history of surgery within the last 30 days.
- Prior hospitalisation within the last 90 days.
- Chronic haemodialysis.
- Residence in a long-term care facility within the last 12 months.

Patients whose first positive *S. aureus* blood cultures were obtained from blood drawn after 48 h of admission were classified as having nosocomially acquired SAB.

Persistent bacteraemia was defined as a positive blood culture for *S. aureus* that was taken more than 72 h after appropriate antibiotics were commenced. Within a patient’s hospital admission, recurrent bacteraemia was defined as a return to *S. aureus* blood culture positivity after an intervening period of documented negative blood cultures and after completing a course of appropriate anti-staphylococcal therapy.

Complicated SAB was defined by the presence of persistent bacteraemia, endocarditis or metastatic infection including septic pulmonary emboli, empyema, staphylococcal pneumonia, osteomyelitis, septic arthritis, staphylococcal bacteriuria, intracerebral space-occupying lesions and deep-seated abscess or meningitis.

Completion of the duration of appropriate anti-staphylococcal antibiotics as recommended by the Infectious Diseases (ID) team was considered ‘adequate’. This was most often 14 days following a negative blood culture in uncomplicated MSSA and MRSA cases and 4 weeks – 6 weeks following a negative blood culture in complicated MSSA and MRSA cases.

Patient information was entered into Microsoft Excel. Descriptive statistics were used to describe demographic and clinical characteristics of the study participants. Frequencies with percentages were used to describe categorical variables. Continuous variables were described by means with standard deviation for normally distributed data or medians with interquartile ranges for continuous variables when skewed. The Shapiro–Wilk test was used to test for normality of continuous variables. The Pearson chi-squared test was used to test for associations between categorical variables or Fisher’s exact when data were sparse (*n* < 5 observations). A logistic regression model was fitted to determine factors associated with death among SAB cases. Covariates were selected *a priori* for model building on the basis of data from previous research on similar patient populations. Statistical significance was set at an alpha level of 0.05. The regression analysis was performed using STATA version 15 (StataCorp, TX).

The study was approved by the Human Research Ethics Committee at a University in SA.

### Ethical considerations

Ethical clearance to conduct this study was obtained from the Human Research Ethics Committee (Medical) at the University of the Witwatersrand, Johannesburg (M221033).

## Results

**FIGURE 1:**
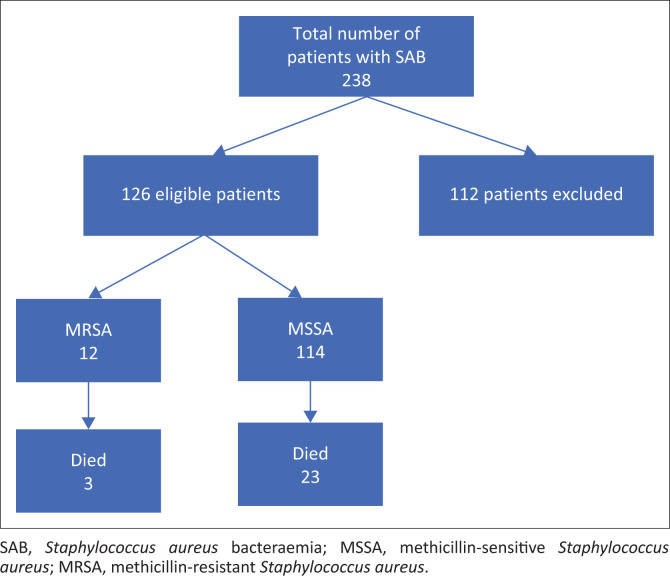
Flow chart of screened and included patients with *Staphylococcal aureus* bacteraemia with designation into methicillin drug sensitivity profile and mortality thereof.

From October 2015 to November 2022, 238 patients with SAB were identified, and 126 patients were included for analysis (Figure 1). A total of 112 patients were excluded as their outcome data were missing. Of the patients included in the study, 26 cases died, representing an in-hospital case fatality ratio of 20.6% (95% confidence interval [CI]: 13.9–28.8).

Case fatality ratios were 20.2% and 25.0% among cases infected with MSSA and MRSA, respectively; however, the differences in mortality rates by strain were not statistically significant (Fischer’s exact *p*-value = 0.154).

The median patient age was 44 years. Males comprised nearly 70% of the cases. As seen in Table 1, the most common mode of acquisition was community acquired (49.2%), followed by nosocomial (32.5%) and lastly healthcare-associated community onset (18.3%). Fifty out of 126 patients were HIV positive while six out of 126 patients had an unknown status. The median CD4 count in this group was low at 79 cells/µL. The most common non-infectious comorbidity was hypertension (26.2%), followed by renal dysfunction, diabetes and neurocognitive or psychiatric disorders. Most patients (73.8%) completed antibiotics with a median antibiotic duration period of 14 days while 18.3% did not complete antibiotics because of death, refusal of hospital treatment and palliation. The median albumin in our study population was low at 30 g/L as shown in Table 1.

**TABLE 1:** Demographic, clinical characteristics, outcomes and risk factors of 126 *Staphylococcus aureus* bacteraemia cases at Helen Joseph Hospital, 2015-2022.

Characteristic	*n* (*N* = 126)	%	Median	IQR	Mean ± s.d.
**Demographics**					
**Age (years)**	-	-	44	34-58	-
< 30	17	13.49	-	-	-
30–39	35	27.77	-	-	-
40–49	25	19.84	-	-	-
50–59	19	15.07	-	-	-
60+	30	23.81	-	-	-
**Sex**					
Female	38	30.16	-	-	-
Male	88	69.84	-	-	-
**Mode of acquisition**					
Community acquired	62	49.21	-	-	-
Healthcare associated	23	18.25	-	-	-
Nosocomial	41	32.54	-	-	-
**Infectious comorbidities (*n* = Yes)**					
**Human immunodeficiency virus (HIV) status**					
HIV-positive cases	50	39.68	-	-	-
HIV-negative cases	70	55.56	-	-	-
Unknown HIV cases	6	4.76	-	-	-
CD4 count cells/μL†	-	-	79	20-255	-
Log10 viral load‡	-	-	4.9	2.7-5.3	-
Active tuberculosis	11	8.73	-	-	-
Hepatitis C	9	7.14	-	-	-
Other	23	18.25	-	-	-
**Non-infectious comorbidities (*n* = Yes)**	-	-	-	-	-
Hypertension	33	26.19	-	-	-
Acute kidney injury	26	20.63	-	-	-
Chronic kidney disease	23	18.25	-	-	-
Diabetes	21	16.66	-	-	-
HbA1c	-		-	-	10.9 ± 3.4
eurocognitive or psychiatric disorders	13	10.31	-	-	-
Trauma	9	7.14	-	-	-
Cardiac failure	8	6.35	-	-	-
Malignancy	8	6.35	-	-	-
Other	38	30.16	-	-	-
**Antibiotic therapy**					-
Completed	93	73.81	-	-	-
Median antibiotic duration for those who completed antibiotics (days) (IQR)	-	-	14	14-15	-
Did not complete	23	18.25	-	-	-
Dead	13	56.52	-	-	-
Refusal of hospital treatment	7	30.43	-	-	-
Palliative	1	4.34	-	-	-
Reason not documented	2	8.70	-	-	-
Median antibiotic duration days (IQR) for those who did not complete antibiotics	-		8	4-10	
Completion of antibiotics unknown (missing data)	10	7.94	-	-	-
**Laboratory parameters, *n*, mean ± s.d. or median (IQR) on admission**	-	-	-	-	-
Haemoglobin (g/dL)	-	-	-	-	10.8 ± 3.1
White blood cell count (× 10 g/L)	-	-	12.5	7.9-16.4	-
Platelets (× 10 g/L)	-	-	247	149-350	-
Creatinine (µmol/L)	-	-	112	72–237	-
Urea (mmol/L)	-	-	9.8	5.4–17.0	-
C-reactive protein (mg/L)	-	-	165	69.5–259.5	-
Albumin (g/L)	-	-	30	24–36	-
**Predisposing factors for developing SAB (*n* = Yes)**					
Previous hospitalisation within the past 90 days	35	27.80	-	-	-
Presence of an invasive device	33	26.20	-	-	-
Short-term central venous catheter	26	78.78	-	-	-
Arterial line	1	3.03	-	-	-
Intercostal drain	3	9.09	-	-	-
Metallic mitral valve	2	6.06	-	-	-
Orthopaedic hardware	1	3.03	-	-	-
Undergoing haemodialysis	19	15.10	-	-	-
Intravenous drug use	12	9.50	-	-	-
Intensive care unit admission	6	4.80	-	-	-
Residence in a long-term care in facility	5	4.00	-	-	-
Recent surgical procedure within past 30 days	4	3.20	-	-	-
**Outcome**					
Dead	26	20.60	-	-	-
Discharged	100	79.40	-	-	-

s.d., standard deviation; HbA1C, glycated haemoglobin A1C; IQR, interquartile range; ARDS, acute respiratory distress syndrome; SAB, *Staphylococcus aureus* bacteraemia.

† , *n* = 49 among HIV-positive patients;

‡ , *n* = 45 among HIV-positive patients.

The most common predisposing factors for developing SAB were previous hospitalisation within 90 days (27.8%) followed by the presence of an invasive device (26.2%), haemodialysis (15.1%) and intravenous drug use (9.5%).

The potential sources of infection in order of prevalence were skin and soft tissue (30.95%), unknown source (26.2%), short-term central venous catheter (20.6%), intravenous drug use (9.5%), other (7.1%) and peripheral venous catheter (5.6%). This clustered sharply by SAB subtype (Table 2). The majority of community-acquired infections were caused by skin and soft tissue infections, while most healthcare-associated and nosocomial-related infections were caused by short-term central venous catheters.

**TABLE 2:** Potential sources of infection among *Staphylococcus aureus* cases at Helen Joseph Hospital, 2015-2022.

Potential sources of infection in the mode of acquistion subtypes	*n*	%
**Potential source of infection in community-acquired cases (*n* = 62)**		
Skin and soft tissue	28	45.20
Intravenous drug use	11	17.70
Other	3	4.80
Unknown	20	32.26
**Potential source of infection in healthcare-associated community-onset cases (*n* = 23)**		
Short-term central venous catheter	10	43.50
Peripheral venous catheter	1	4.30
Skin and soft tissue	5	21.70
Intravenous drug use	1	4.30
Unknown	6	26.10
**Potential source of infection in nosocomial cases (*n* = 41)**		
Short-term central venous catheter	16	39.00
Peripheral venous catheter	6	14.60
Skin and soft tissue	6	14.60
Other	6	14.60
Unknown	7	17.10

Most high-income resource settings recommend screening all SAB patients with an echocardiogram, preferably a TEE.^7,8^ However, this may not be feasible in a low-resource setting where echocardiograms are a scarce resource. Coming from a middle-income tertiary setting, our facility was able to screen most SAB patients for IE with at least a transthoracic echocardiogram (TTE). A total of 107 out of 126 patients (84.9%) were evaluated for infective endocarditis (IE) with an echocardiogram – 100 by TTE, six with a transoesophageal echocardiogram (TOE) and only one patient had both a TOE and a TTE. The remaining 19 patients did not have an echocardiographic evaluation because of death or absconding from treatment and/or care. The prevalence of IE among cases of SAB in the study sample was 16/107 (15%). Most of the IE cases were diagnosed using a TTE (6/16), only two were diagnosed using TOE and one was diagnosed using both TOE and TTE, while in 7/16 cases it was not clear which type of echocardiogram was used.

The number of patients with at least one complication was 28 (22.2%, Table 3). Metastatic infections were largely correlated with the IE. In our study, 11/16 IE cases were community acquired, 4/16 cases were healthcare-associated community onset and only one case was nosocomially acquired (*p* = 0.08).

**TABLE 3:** Prevalence of complications associated with *Staphylococcus aureus* bacteraemia in the study sample.

Complication	*N*	%
Infective endocarditis	16	15.0
Metastatic infection	15	11.9
Septic pulmonary emboli	6	40.0
Bacteriuria	3	20.0
Other	6	40.0
Osteomyelitis	2	1.60
Persistent bacteraemia	1	0.80
Recurrence	0	-

In the univariate analysis, the odds ratio of dying was significantly higher among cases of SAB aged 50 years and older compared to their younger counterparts (< 50 years of age), odds ratio, OR: 2.65 (95% CI: 1.10–6.39). Similarly, cases of SAB with hypertension and those with a history of hospital admission in the previous 3 months had a higher risk of dying in the unadjusted model (Table 4).

**TABLE 4:** Logistic regression model demonstrating factors associated with death among patients with *Staphylococcus aureus* bacteraemia at Helen Joseph Hospital.

Variable	*N* †	%	Unadjusted odds ratio	95% CI	*p*	Adjusted odds ratio	95% CI	*p*
**Age group (years)**	-	-	-	-	0.030*	-	-	0.598
< 50	11/77	14.3	1.00	-	-	1.00	-	-
50+	15/49	30.6	2.65	1.10-6.39	-	0.68	0.17–2.81	-
**HIV status**	-	-	-	-	0.228	-	-	0.188
Negative	16/70	22.9	1.00	-	-	1.00	-	-
Positive	7/50	14.0	0.55	0.21-1.46	-	0.41	0.11–1.54	-
**Acute kidney injury**	-	-	-	-	0.157	-	-	0.056
None	18/100	18.0	1.00	-	-	1.00	-	-
Yes	8/26	30.8	2.02	0.76-5.38	-	3.56	0.93–13.56	-
**Diabetes mellitus**	-	-	-	-	0.844	-	-	0.150
No	22/105	21.0	1.00	-	-	1.00	-	-
Yes	4/21	19.1	0.89	0.27-2.91	-	0.29	0.05–1.56	-
**Hypertension**	-	-	-	-	0.012*	-	-	0.020*
No	14/93	15.1	1.00	-	-	1.00	-	-
Yes	12/33	36.4	3.22	1.30-8.00	-	5.55	1.31–23.55	-
**Cardiac failure**	-	-	-	-	0.563	-	-	0.370
No	25/118	21.2	1.00	-	-	1.00	-	-
Yes	1/8	12.5	0.53	0.06-4.52	-	0.27	0.02–4.76	-
**SAB complications**	-	-	-	-	0.906	-	-	0.665
No	20/98	20.4	1.00	-	-	1.00	-	-
Yes	6/28	21.4	1.06	0.38-2.97	-	0.74	0.19–2.94	-
**ICU admission**	-	-	-	-	0.806	-	-	0.697
No	25/120	20.8	1.00	-	-	1.00	-	-
Yes	1/6	16.7	0.76	0.08-6.80	-	1.68	0.12–23.01	-
**Intravenous drug use**	-	-	-	-	0.623	-	-	0.867
No	24/113	21.2	1.00	-	-	1.00	-	-
Yes	2/13	15.4	0.67	0.14-3.25	-	0.89	0.13–5.72	-
**Long-term facility care**	-	-	-	-	0.971	-	-	0.390
No	25/121	20.7	1.00	-	-	1.00	-	-
Yes	1/5	20.0	0.96	0.10-8.97	-	0.27	0.01–5.39	-
**Use of invasive device**	-	-	-	-	0.676	-	-	0.169
No	19/96	19.8	1.00	-	-	1.00	-	-
Yes	7/30	23.3	1.23	0.46-3.30		3.97	0.56–28.28	-
**Undergoing haemodialysis**	-	-	-	-	0.961	-	-	0.175
No	22/107	20.6	1.00	-	-	1.00	-	-
Yes	4/19	21.1	1.03	0.31-3.42	-	0.22	0.03–1.95	-
**Recent surgical procedure within 30 days**	-	-	-	-	0.134	-	-	0.527
No	15/88	17.1	1.00	-	-	1.00	-	-
Yes	11/38	29.0	1.98	0.81-4.85	-	0.57	0.10–3.24	-
**Hospitalisation ≤ 90 days**	-	-	-	-	-	-	-	0.009**
No	13/91	14.3	1.00	-	0.006**	1.00	-	-
Yes	13/35	37.1	3.55	1.44-8.74	-	11.88	1.84–26.99	-
**Infection sub-types**	-	-	-	-	-	-	-	0.134
Community acquired	11/62	17.7	1.00	-	-	1.00	-	-
Healthcare associated	8/23	34.8	2.47	0.84-7.23	0.100	0.50	0.05–4.63	-
Nosocomial	7/41	17.1	0.95	0.34-2.71	0.930	0.20	0.03–1.26	-

CI, confidence interval; ICU, intensive care unit; SAB, *Staphylococcus aureus* bacteraemia; HIV, human immunodeficiency virus.

* , significant difference at *p* < 0.05;

** , significant difference at *p* < 0.01.

† , Number with outcome out of total number of cases.

In the multivariate analysis, having hypertension (OR: 5.55 [95% CI: 1.31–23.55]) and having been hospitalised in the previous 3 months (OR: 11.88 [95% CI: 1.84–26.99]) were independently associated with dying among cases of SAB at a tertiary-level hospital in SA. Patients with acute kidney injury (AKI) had 3.6 times higher odds of dying compared to patients who did not, adjusted odds ratio 3.56 [95% CI: 0.93–13.56]; however, statistical significance was borderline (*p* = 0.056).

## Discussion

Our study found an all-cause SAB case fatality ratio of 20.6%. This is comparable to the case fatality ratio seen in other studies from high-income settings, which ranges from 20% to 30%.^3,4,9,10,11^ suggesting that similar outcomes are possible in a middle-income setting, despite more limited resources. Of note, the ID division routinely examines all SAB cases in HJH, offering advice on aspects of care such as antibiotic choice, treatment duration, source control and the need for echocardiography. An ID consultation has been associated with lower mortality from SAB in several studies from high-income settings.^1^ Supporting the role of similar consultations in a middle-income setting, one prospective observational study conducted in Groote Schuur Hospital in 2018 found an inpatient 90-day mortality of 47% but a more recent study in Groote Schuur Hospital had an inpatient mortality rate of 16% after the implementation of an ID specialist-led standardised bundle of care recommendations on its 86 patients.^2,4^

The relatively low rate of MRSA in our cohort may also have contributed to the lower-than-expected case fatality ratio, as MRSA has been associated with a higher risk of death compared to MSSA in several studies.^12^

Other studies have found age to be associated with SAB mortality.^13^ Although age > 50 years was associated with mortality in the crude analyses in our study, this association was not found to be statistically significant in the adjusted multivariate analyses. Our study population had a relatively young median age at 44 years possibly because of the burden of HIV.^3^

The impact of HIV on SAB has not been well-described, either as a risk factor in acquiring the disease or as a predictor of mortality. Although our study could not determine whether HIV was a risk factor for SAB, the prevalence of HIV in the study population (40%) was almost exactly the same as the prevalence of HIV in the general medical hospital wards at HJH.^14^ In our study, HIV was also not a risk factor for death among SAB patients (OR: 0.41; 95% CI: 0.11–1.54, *p* = 0.188).

The male-to-female ratio in this study was 2.3:1, which was compatible with the higher incidence of SAB seen for males in the existing literature.^3,5^ Possible reasons for why gender may play a role in outcomes include higher risk-taking behaviour adopted by men leading to skin breaks or the higher proportion of intravenous drug use in men.^12^

The most common comorbidities in patients with SAB in this study seem to be somewhat similar to other studies – HIV infection (39.7%), renal dysfunction (38.9%), hypertension (26.2%), diabetes (16.7%) – with the exception of neurocognitive and/or psychiatric disorders (10.3%), which has not been reported in other literature to date.^3,15,16^ Most of the patients with neurocognitive and/or psychiatric disorders were long-term residents in healthcare facilities, which was a predisposing factor for developing SAB in this study.

Interestingly, this study identified slightly different risk factors for in-hospital mortality compared to other studies, namely prior hospitalisation within 90 days (*p* = 0.005), hypertension (*p* = 0.034) and AKI (*p* = 0.054). Possible reasons for recent hospitalisation being a risk factor for in-patient mortality include recent exposure to central and peripheral venous catheters and delayed diagnosis because of overlap of SAB symptoms with symptoms of their recent illness or comorbidities. Similarly, renal dysfunction was also associated with in-patient mortality on the multivariate analysis. Renal failure is often associated with a higher inpatient mortality in many conditions, and confounding factors may include concomitant nephrotoxins and inappropriate pharmokinetics because of fluctuating renal function as a consequence of the disease itself.^12^ The reason for hypertension’s association with mortality is unclear, and this association has not featured strongly in the literature. As the lower border of the 95% confidence interval was 1.12, it is possible that chance may still explain this association. Alternatively, residual confounding with other comorbidities may still be present.

The majority of the infective episodes were community acquired (49.2%) and related to skin and soft tissue infections. This is higher than that seen in a recent retrospective study based at Groote Schuur Hospital, which found that 71% of cases with SAB were hospital associated versus only 25% acquired in the community.^2^ Similar to other settings, almost 50% of our cases were healthcare associated and nosocomially acquired and were largely related to short-term central venous catheters. This suggests that many may have been preventable with better care to central venous catheter hygiene, highlighting the need for improved infection prevention practices.^4^

Community-acquired MRSA was very rare in our cohort (3.17%) compared to 20% of community-acquired MRSA cases seen in a retrospective review of 449 participants in two academic hospitals in SA.^3^ Our study did not find a statistically significant mortality rate between MSSA and MRSA cases (*p* = 0.154).^3^ This could be because of the small number of MRSA cases identified, which did not provide sufficient power for statistical comparison. Interestingly, the vast majority (87.5%) of nosocomial and healthcare-associated cases in our study were MSSA bacteraemias, which are in contrast to international literature where up to 50% were MRSA bacteraemias.^10,11^ This finding has potentially important implications for empirical antimicrobial therapy for inpatients who develop *S. aureus* infections, as vancomycin treatment is associated with poorer outcomes than therapy with anti-staphylococcal beta-lactams.^17^

The prevalence of endocarditis among cases of SAB in this study was 15%, which was similar to the proportions of IE cases because of *S. aureus* in one international meta-analysis study, which ranged from 16% to 34%.^13^ A concern among our study population is the possibility that the prevalence of IE reported in our study might have been underestimated, as 99% of the patients were examined with the less-sensitive TTE without an additional TOE. Of those patients diagnosed with IE in our study, 8/16 were people who inject drugs that could indicate that these groups are high risk and should be prioritised for an echocardiogram. Intensive care unit admission, valvular heart disease (although there were very few of these in this study) and being previously hospitalised within 90 days were not associated with IE. In our study, the case fatality ratio was 18.8% (95% CI: 4.0–45.6) among patients with IE versus 14.3% (95% CI: 7.8–23.2) among patients without IE; however, this difference was not statistically significant (Fischer’s exact *p*-value = 0.704). In contrast to international studies that see a much higher prevalence of healthcare-associated IE, in our study, 68.8% of IE cases were community-acquired.^13^

Fewer than a quarter of the study patients (22.2%) had a complicated infection. In keeping with previous reports, this rate was higher in the community-acquired subset (63.6%), presumably because of delayed presentation and treatment.^4,13^

In our study, there was only one case of persistent bacteraemia. In this case, the patient was readmitted for a septic arthritis that was not timeously surgically drained following recent hospital admission for heart failure. There were no cases of recurrent bacteraemia in this study. Possible reasons for this include early diagnosis, prompt and appropriate antibiotic cover, fast source control and guidance of the ID specialist team.

Our study has several important limitations. The study was retrospective and attributable mortality because of SAB could not be directly inferred. Another important limitation was that patients who died during the period between their blood culture draw and provisional laboratory identification of *S. aureus* on that blood culture would not have been seen by the ID service, and thus the case fatality ratio in this study likely represents an underestimate of the true mortality burden. The extent of this is not possible to quantify from our data, but it is mitigated by the fact that the IDs team is made aware of all possible *S. aureus* bacteraemias by the laboratory prior to final laboratory confirmation. A further limitation to our study is that patients in this study all had a consultation by ID specialists and majority had an echocardiogram, limiting generalisation of the outcome data as not all hospitals in the region have such support. Additionally, many patients were excluded as the outcome was missing. Of covariates adjusted for in this model, the overall fitted model was not statistically significant (overall *p*-value for the model = 0.141), suggesting that the covariates adjusted for did not fully explain mortality in this sample. Potential reasons include a small study sample, rendering the study less powerful or some other residual confounder.

## Conclusion

Despite a substantial SAB-associated mortality, the case fatality ratio in our setting is comparable to higher-income countries, suggesting that acceptable outcomes are achievable in tertiary middle-income settings, provided that there is access to the resources available in our setting, including infectious diseases consultation, echocardiograms and basic infection control practices.

In contrast to other local and international studies, MRSA was not significantly prevalent in our study and the vast majority of our nosocomial and healthcare-associated cases were MSSA. This has potentially important implications for empiric antibiotic coverage in such patients.
